# Playing with fire – What is influencing horse owners’ decisions to not vaccinate their horses against deadly Hendra virus infection?

**DOI:** 10.1371/journal.pone.0180062

**Published:** 2017-06-21

**Authors:** Kailiea Arianna Goyen, John David Wright, Alexandra Cunneen, Joerg Henning

**Affiliations:** School of Veterinary Science, Faculty of Science, University of Queensland, Gatton, Queensland, Australia; University of Minnesota, UNITED STATES

## Abstract

Hendra virus is a zoonotic paramyxovirus, which causes severe respiratory and neurological disease in horses and humans. Since 2012, the Hendra virus sub-unit G vaccine has been available for horse vaccination in Australia. Uptake of the vaccine has been limited and spill-over events of Hendra virus infection in horses continue to occur. We conducted an online, questionnaire-based cross-sectional study of 376 horse owners belonging to a variety of different equestrian clubs in Queensland, Australia, to identify risk factors for non-vaccination against Hendra virus. A total of 43.1% (N = 162) of horse owners indicated that they currently did not vaccinate against Hendra virus infection, while 56.9% (N = 214) currently vaccinated against Hendra virus infection. A total of 52 risk factors were evaluated relating to equestrian activities, horse management, perceived risk and severity of horse and human infection with Hendra virus, side effects of Hendra vaccination, other vaccinations conducted by horse owners and horse owners’ attitudes towards veterinarians. The final multivariable logistics regression model identified the following risk factors associated with increased odds of non-vaccination against Hendra virus: 1) perceived low risk (compared to high) of Hendra virus infection to horses (considering the horse owners’ location and management practices) or horse owners were unsure about the risk of infection, 2) perceived moderate severity (compared to very severe or severe) of Hendra virus infection in humans, 3) horse owners non-vaccination of their pets, 4) horse owners non-vaccination against strangles disease in horses, 5) handling of more than three horses per week (compared to one horse only) and 6) perceived attitude that veterinarians had a high motivation of making money from Hendra virus vaccination (compared to veterinarians having a low motivation of making money from Hendra virus vaccination). Horse owners were more likely to vaccinate against Hendra virus if horses were used for dressage, show jumping or eventing. The study also identified horse owners’ concerns about side-effects and about the lack of evidence on vaccine efficacy.

## Introduction

Hendra virus (HeV) is a zoonotic paramyxovirus first recognised in 1994 [1–3]. Since its emergence HeV has caused sporadic outbreaks of respiratory and neurological disease characterised by significant mortality [3]. Pteropid fruit bats are the natural reservoir of the virus, [4] with spill-over events into horses, horse to horse and horse to human transmission occurring [5]. Whilst HeV infection in horses has generally been associated with respiratory and neurological signs, clinical signs can be non-specific, which can be mistaken for common diseases such as colic, pneumonia or pleuropneumonia [6–7]. Viral shedding may occur prior to clinical disease, which is a concern because of the risk of horse to human transmission, and an issue for veterinarians attending these animals [8–9].

HeV is maintained in four species of mainland Australian flying fox; these species are known as the black flying fox (*Pteropus alecto)*, grey-headed flying fox (*P*. *poliocephalus)*, little red flying fox *(P*. *scapulatus)*, and the spectacled flying fox *(P*. *conspicillatus)* [6, 10]. The majority of spill-over events into horses have occurred along the eastern coast of Queensland and the north-east coast of New South Wales [6]. The rate of spill-over events from flying foxes is considered to be low, [4] and is thought to occur when horses are exposed to flying fox secretions [8]. The exact mode of horse to human transmission is unknown but is proposed to be from direct contact with infected equine secretions, fluids, tissues, droplets or aerosols [6]). The current attack rate for humans exposed to infected secretions and fluids is low and estimated to be approximately 10% [8]. Although not highly contagious, the consequences are severe as the mortality rate is high, currently being 57% [6, 11].

The factors relating to transmission and spill-over into horses are currently not fully understood and it is likely that multiple factors are involved [12–13]. Management instructions provided to horse owners to reduce the risk of HeV infection in horses are complex and potentially difficult to implement (e.g. reducing canopy density, covering water containers at night)–thus there is no simple protocol to prevent horses coming into contact with HeV and becoming infected [9, 12–13]. Consequently a substantial proportion of horse owners do not implement appropriate risk mitigation strategies [11]. Veterinarians have a legal liability in relation to providing a safe workplace (as stated in Queensland Veterinary Surgeons Act of 1936, Section 28 of the Queensland Workplace and Safety Act). Thus the legal liability and the occupational risk of HeV are of concern to veterinarians and an increasing number have withdrawn from equine practice [14].

There are no currently registered therapies available for the treatment of HeV infection in horses or humans. All HeV infected horses are euthanised once diagnosis is confirmed [6]. Monoclonal antibodies, antiviral drugs, peptide-based fusion inhibitors and receptor blockaders are being explored for human use [7, 15]. The HeV subunit-G vaccination was developed to protect horses against HeV and therefore indirectly, reduce the risk of horse to human transmission [9]. The vaccine was released in 2012 with a minor use permit [6] and was registered in August 2015 [16]. The initial uptake of the vaccine has been limited [11], with the highest uptake occurring in coastal north-east NSW and Queensland, which are designated as the areas of greatest apparent risk [6]. Proposed factors for poor vaccine uptake include horse owner risk assessment, safety and efficacy issues, and lack of evidence of safety in pregnant mares, export restrictions and cost of vaccination [9]. Spill-over events have continued since the release of the vaccine [12]. The vaccine has been shown to be highly effective at 21 days and 6 months post vaccination [9], but no studies have been published on long term efficacy, which has meant that initial vaccination schedule required six monthly boosters. The current vaccination schedule (from May 2016) requires two doses between three and six weeks apart, an initial 6-month booster followed by annual boosters thereafter [16–17].

There are no published data in scientific journals on the short or long term side-effects of the vaccine, nor its effects on breeding animals. However, the *Australian Pesticides and Veterinary Medicine Authority* has collected data on adverse experience with the vaccine as part of its ongoing surveillance of the registered product. This information was received under a mandatory reporting regimen which was required as a condition before the registration of the product, and a summary of adverse experience reports made to the APVMA about Hendra virus vaccine was released in September 2016 [18]. This has enabled the HeV vaccine to be approved for pregnant mares (after day 45 of gestation and before 14 days of expected foaling) and used in foals >4 months of age.

The objective of this study was to identify factors that influence horse owners in Queensland who keep, breed or use horses for different purposes to not vaccinate their horses against HeV. Thus we investigated the association between HeV non-vaccination and horse owners concerns and attitudes towards HeV vaccine, perceptions of risk and severity of HeV, and horse owners’ attitudes towards the group of professionals who deliver the vaccine (i.e. veterinarians).

## Materials and methods

### Study design

This study was an online, questionnaire-based, cross-sectional study. Queensland horse owners were the target population for the study. As no databases of horse ownership are publicly available, the survey was directed at horse owners who belonged to equestrian clubs. Equestrian Australia or Pony Club Associations or other Equestrian Clubs in Queensland, Australia, were identified through internet searches. A total of 396 Queensland horse clubs and associations were identified and contacted using the email addresses specified on the clubs’ and associations’ websites. Club secretaries were asked to disseminate the link to an online survey to their members. Club secretaries contacted their members per email—email communication is the normal method for clubs to maintain contact with their members. The authors of this research paper had no access to lists of members and therefore members were not identifiable from the survey. We also asked the equestrian clubs to specify the number of members to whom emails were sent out with the link to the online survey.

Human Ethics approval for this research was obtained from the University of Queensland, Australia (Approval number: 2015000863).

### Questionnaire

The questionnaire consisted of 94 questions. These questions captured demographic and property information, horse husbandry and management practices, vaccination rates for HeV, strangles and tetanus, rates of other vaccinations (self, children, pets), perceived risk and severity of tetanus, strangles and HeV infections in horses, perceived risk and severity of human HeV infection, side effects of HeV, strangles and tetanus infection and horse owner’s attitudes towards veterinarians, who conduct the HeV vaccinations. The majority of questions were comprised of multiple choice and questions on a 4 or 5 point Likert scale. Open-ended questions were also included in the questionnaire and explored horse owners’ opinions on improving HeV vaccination uptake. The questionnaire was revised based on comments provided by clinical academics working with horses. The questionnaire was pilot-tested with 10 horse owners representing various equestrian activities and approximately 15 questions were revised. The questionnaire could be completed within 15 minutes. The questionnaire was delivered via the online platform ‘SurveyMonkey’. The survey was open for the period 27 July 2015–29 September 2015, with two reminders emailed in August and September prior to closure of the survey. To determine a response rate clubs were asked to provide the number of members who had been emailed with the link to the questionnaire.

### Data analysis

Correlation between risk factors was assessed by developing a matrix plot and by calculating pairwise Pearson correlations. If high bivariate correlations (Pearson correlation coefficient > 0.7) were present, one of the two variables was not included in the further analysis.

Associations between potential risk factor variables and the decision of horse owners not to vaccinate against HeV were assessed using logistic regression models. Maximum-likelihood logistic regression models were fitted using Stata version 14.0 (StataCorp, USA). Overall significance of variables was assessed using likelihood ratio P values. Significance of individual levels for risk factors with more than two levels (relative to the reference level) was assessed using Wald test. After the univariate analysis, all variables that had overall likelihood ratio p-value or Wald test p-value of <0.2 were included in the multivariable modelling. Each of the variables was fitted using a forward selection with variables sequentially fitted in ascending order based on likelihood ratio p-values. Any variable that resulted in a chance of coefficients of more than 20% of variables already in the model remained in the model. A backward selection approach with variables sequentially removed based on P values<0.05 was also conducted. Meaningful first-order interaction terms were also tested.

The fit of the final maximum-likelihood logistic model was assessed using the Hosmer–Lemeshow goodness-of-fit test and the discriminatory ability of the final model was assessed using the area under the receiver operator characteristics (ROC) curve.

## Results

The survey was completed online by 384 respondents. Exclusions of respondents were applied to those only partially completing the questionnaire (8); a total of 376 respondents were used for the analysis. A total of 95.2% of participants were female, 4% were male and 0.8% refused to disclose their gender. It was not possible to calculate a response rate as clubs did not provide numbers of members contacted.

### Risk factors for non-vaccination against HeV infection

A total of 43.1% (N = 162) of horse owners belonging to equestrian clubs indicated that they currently did not vaccinate against HeV infection, while 56.9% (N = 214) currently vaccinated against HeV infection.

**Fig 1:**
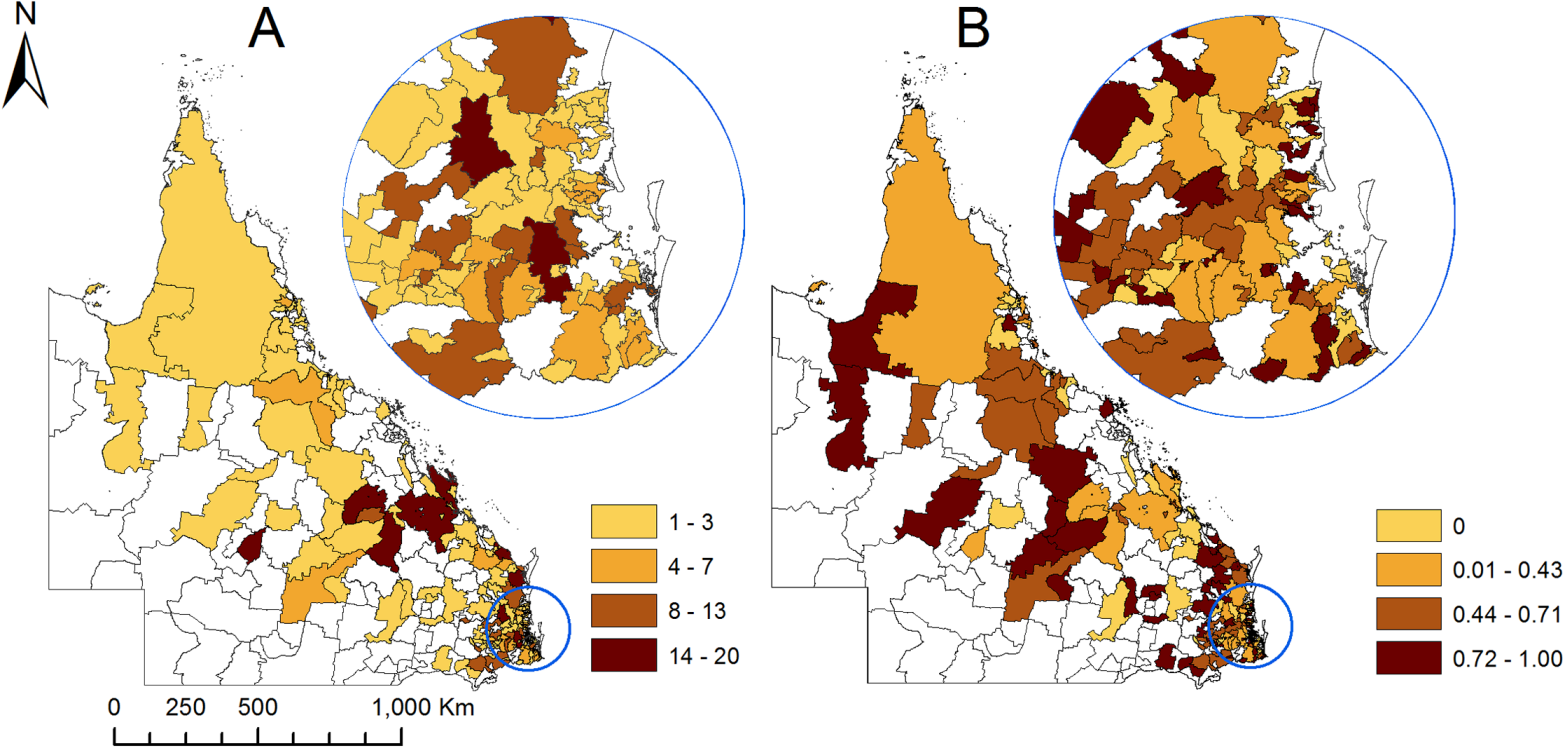
Total number of horse owners surveyed per postcode area (A) and proportion of surveyed horse owners not vaccinating their horses against Hendra virus infection (B) in 2015, in Queensland, Australia.

The proportion of horse owners vaccinating and not vaccinating their horses against Hendra virus in 2015 by postcode area is shown in Fig 1. There were clusters of inland postcode areas, where more than 70% of respondents did not vaccinate against HeV.

Results of the univariate analysis are shown in S1 Table. Of the 52 risk factors evaluated, a total of 32 risk factors were significant at p<0.2 and were included in the multivariable model. The final multivariable model included 8 risk factors (Table 1). Horse owners were more likely not vaccinating against HeV, if 1) the risk of HeV to their horses (considering the horse owners’ location and management practices) was assessed to be low (compared to high) or if horse owners were unsure about the risk of HeV to their horses, 2) the severity of HeV infection in humans was considered to be moderate (compared to very severe or severe), 3) horse owners did not vaccinate their pets, 4) horse owners did not vaccinate their horses for strangles, 5) horse owners handled three or more horses per week on their property (compared to one horse only) and 6) horse owners assessed that veterinarians had a high motivation to ‘make money’ from HeV vaccination (compared to veterinarians having a low motivation to make money from HeV vaccination). Horse owners were more like to vaccinate against HeV if they used their horses for dressage/show jumping/eventing (1/0.34 = 2.97 times more likely to vaccinate against HeV). Veterinarian's motivation to protect horse owners by HeV vaccination was a confounder and remained in the model, although not significant at p<0.05. Removing this variable resulted in a more than 20% change of the coefficients for risk of HeV to horses. Interestingly, horse owners were more likely to vaccinate if veterinarian's motivation to protect horse owners by HeV vaccination was rated as very high (although not significant at p<0.05).

**Table 1:** Results of the multivariate analysis of risk factors associated with non-vaccination of horses against Hendra virus in 2015, in Queensland, Australia.

Risk factor	Level	HeV Vac		No HeV Vac		OR	LCI95%	UCI 95%	p-value	Wald p-value
		N	%	N	%					
Perceived risk of HeV infection in horses	Very high—high	73	88.0	10	12.1	Ref				<0.001
	Low—very low	134	48.0	145	52.0	4.7	2.2	10.1	<0.001	
	I don't know	7	50.0	7	50.0	6.5	1.5	28.1	0.013	
Perceived severity of HeV infection in people	Very severe—severe	208	58.8	146	41.6	Ref				0.063
	Moderate	3	23.1	10	76.9	7.0	1.4	35.7	0.020	
	Mild—very mild	3	33.3	6	66.7	0.9	0.2	4.5	0.862	
Vaccination of horse owner’s pets	Yes	199	60.9	128	39.1	Ref				
	No	15	30.6	34	69.4	2.6	1.1	5.9	0.025	
Currently vaccinate for strangles?	Yes	169	64.5	93	36.1	Ref				
	No	45	39.5	69	60.5	2.0	1.1	3.6	0.017	
Dressage/Show jumping/Eventing	No	83	45.4	100	54.6	Ref				
	Yes	131	67.9	62	32.1	0.3	0.2	0.6	<0.001	
Number of horses owned and frequently handled on property	1	35	79.6	9	20.5	Ref				0.009
	2–3	87	64.9	47	35.1	1.5	0.6	4.1	0.424	
	>3	92	46.5	106	53.5	3.1	1.2	8.2	0.022	
Perceived motivation of veterinarians to conduct HeV vaccination to make money	Unimportant—low important	73	81.1	17	18.9	Ref				0.003
	Neutral	53	67.1	26	32.9	1.6	0.7	3.5	0.280	
	Important—very important	67	38.1	109	61.9	3.5	1.7	7.2	0.001	
	Not Specified	21	67.7	10	32.3	1.2	0.4	3.8	0.716	
Perceived motivation of veterinarians to conduct HeV vaccination to protect horse owners	Unimportant—low important	10	32.3	21	67.7	Ref				0.002
	Neutral	9	20.0	36	80.0	2.6	0.8	8.4	0.107	
	Important—very important	186	66.4	94	33.6	0.5	0.2	1.2	0.117	
	Not Specified	9	45.0	11	55.0	1.0	0.3	3.4	0.995	

There was no evidence of a lack of fit of the final model. The discriminatory ability was fair with the area under the ROC curve being equal to 0.86.

### Education about Hendra virus

There were no significant differences in the level of the self-assessed knowledge about Hendra virus between horse owners vaccinating or not vaccinating against HeV (p = 0.401), with 57.6% of HeV vaccinators indicating to have a very high to high knowledge, 35.6% indicating moderate and 6.8% indicating low to very low knowledge, compared to 46.9%, 44.1% and 9.1% of non-HeV vaccinators, respectively.

**Fig 2:**
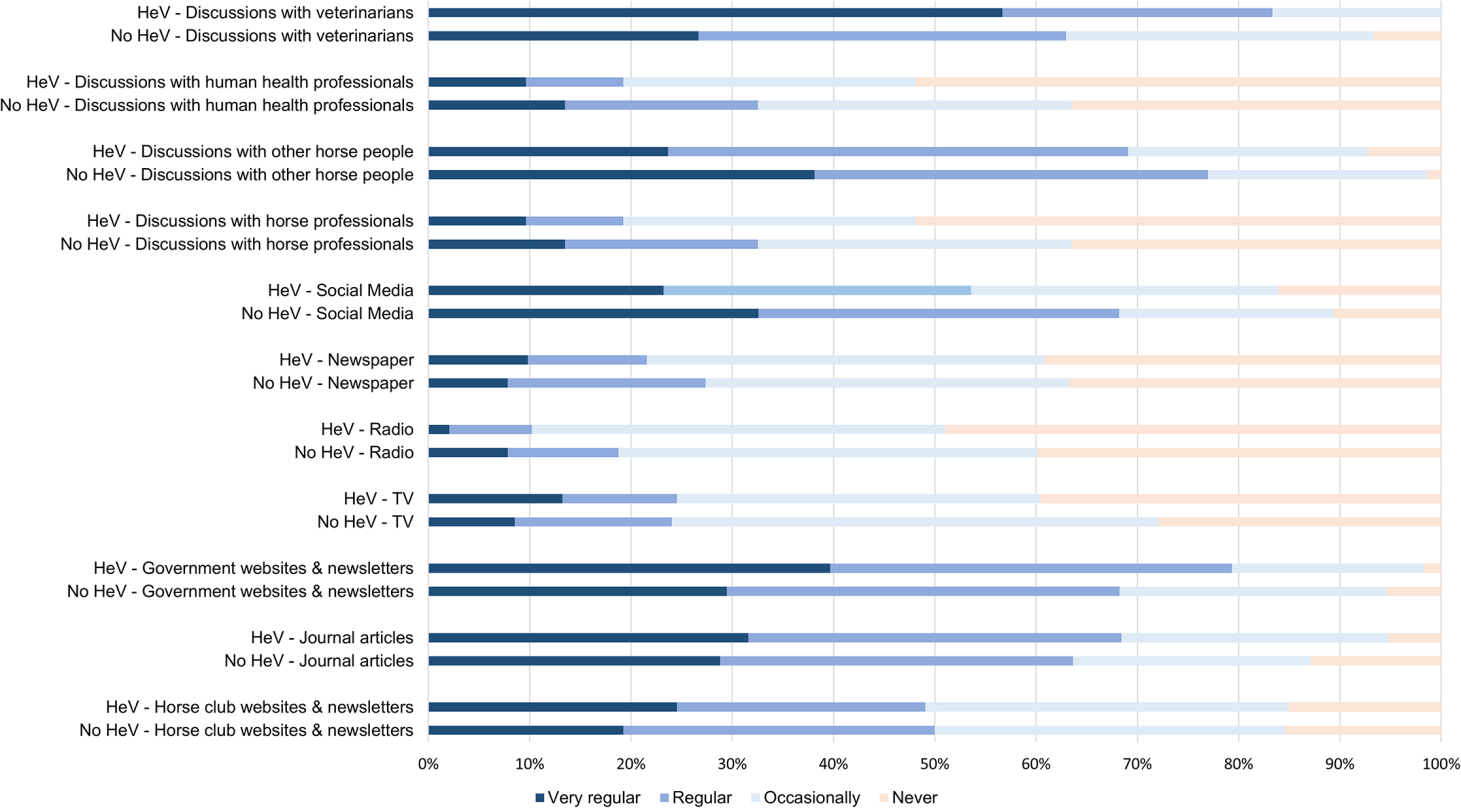
Sources of educational information about Hendra virus infection used by horse owners who were vaccinating and not vaccinating their horses against Hendra virus. Data were collected in 2015, in Queensland, Australia.

When comparing the sources of information that were used by horse owners to inform themselves about Hendra virus (Fig 2), no significant difference at p<0.05 were found between HeV vaccinators and non-HeV vaccinators for the media used (TV, radio, newspapers), newsletters and websites from horse clubs and state or other government bodies and using discussions with horse professionals, horse owners and human health professionals. However, HeV vaccinators are more likely to obtain information about Hendra virus from veterinarians compared to non-HeV vaccinators (p<0.001).

### Motivation for vaccination by non-vaccinators

**Fig 3:**
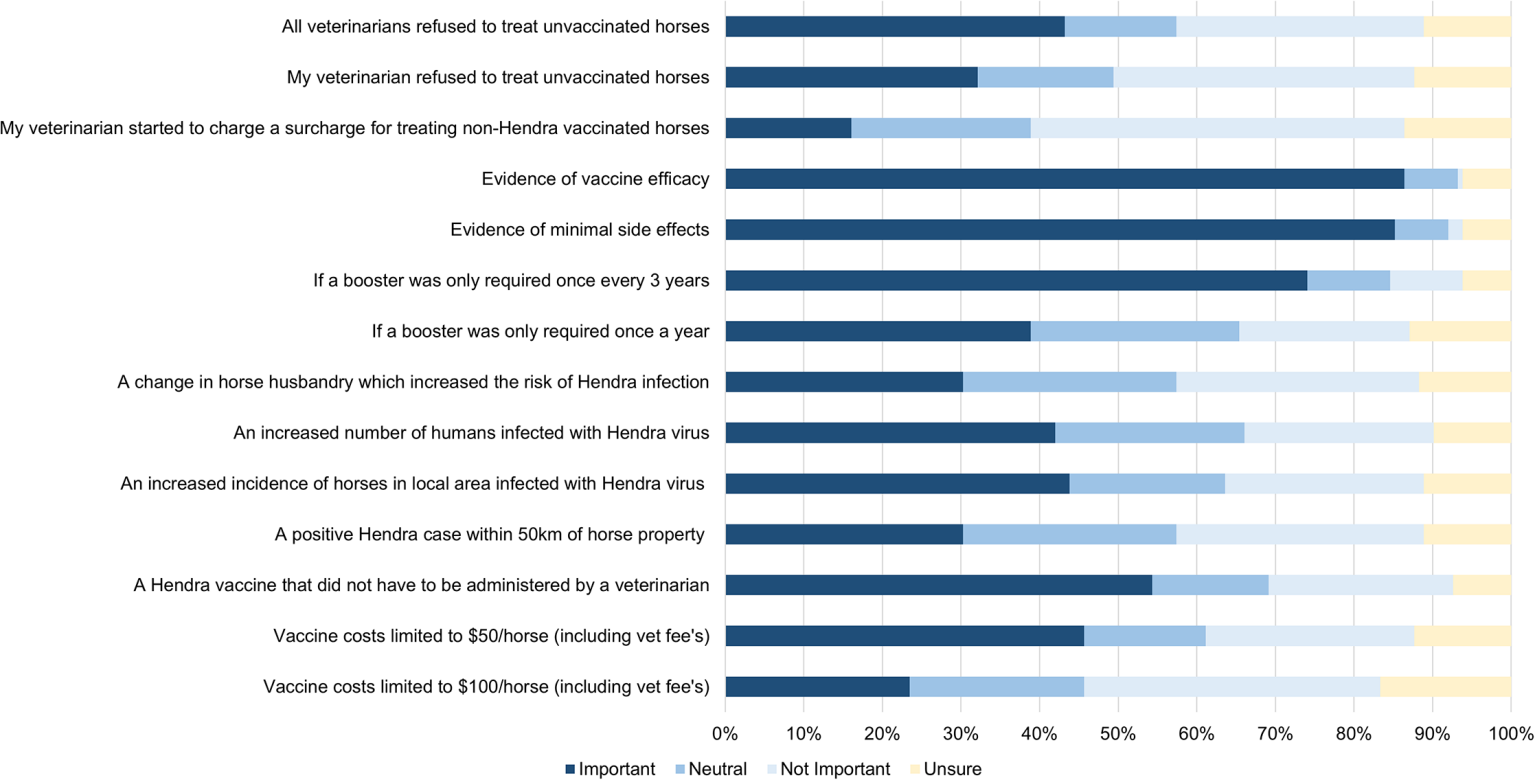
Factors rated as likely to influence non-vaccinating horse owners’ decision to vaccinate their horses against Hendra virus in the future. Data were collected in 2015, in Queensland, Australia.

Fig 3 shows the rating of factors of non-vaccinators that would make them more likely to vaccinate in the future. The most prominent reasons are evidence of vaccine efficacy (86%), evidence of minimal side effects (85%), and change of booster frequency to three yearly (74%).

## Discussion

Almost half of the respondents did not vaccinate against HeV (43.1%, n = 162). Only 16.5% (n = 62) did not vaccinate their horse or horses against tetanus. In the univariate analysis, the risk of HeV non-vaccination was 1.9 times significantly higher (p = 0.018) in those respondents who did not vaccinate their horse or horses against tetanus. Additionally, 30.3% (n = 114) of respondents did not vaccinate their horse or horses against strangles and in the univariate analysis, the risk of HeV non-vaccination was 2.8 times significantly higher (p<0.001) in those respondents who did not vaccinate their horse or horses against strangles.

Tetanus in horses is well-recognised by horse owners as being highly fatal, the vaccine as being highly effective and its administration rarely associated with adverse events; it has also been in use for decades [19]. Of those respondents who vaccinated their horse or horses against tetanus in the current study, 92.6% had not observed any adverse effects. Strangles, on the other hand, is recognised by knowledgeable horse owners as a disease with high morbidity but low mortality, and the vaccine is considered to be effective in reducing the severity of the disease, but does not guarantee prevention [20]. Strangles vaccination is perceived by horse owners as causing not more adverse events than tetanus vaccination (of those respondents who vaccinated their horse or horses against strangles, 92.1% had not observed any adverse effects); again, strangles vaccines have been available for decades. The survey did not ask the question as to whether tetanus and strangles were vaccinated against concurrently. Interestingly, respondents who did not vaccinate against HeV considered strangles infection in unvaccinated horses to be less severe compared to respondents vaccinating against HeV. This result reflects either non-vaccinators excusing their actions or anti-vaccination philosophies by not acknowledging the potential severity of strangles and HeV as diseases, or ignorance by some responders about the potential consequences of disease caused by strangles and HeV in particular. Lack of knowledge about disease severity could lead to non-vaccination as the perception would be that there was no need to vaccinate against agents that do not cause severe disease.

Almost one in five did not vaccinate their horses against tetanus and one in three against strangles. These figures suggest an overall poor uptake of horse vaccinations but the reasons for this were not elucidated in the current survey. Considering these findings, and that many horse owners perceived the HeV vaccine to be experimental, hurriedly introduced and lacking efficacy data, with a high incidence of adverse effects, with unknown effects on horses used for breeding and during pregnancy, and accompanied by the attitude held by a substantial number of horse owners that the risk of HeV infection in horses is low, it is not surprising that many owners have opted not to vaccinate their horses against HeV.

Many more respondents reported adverse effects following HeV vaccination than for tetanus and strangles vaccination, but it is possible that those who observed adverse effects following HeV vaccination were more motivated to reply to the survey, which may have created a potential selection bias. However, the difference between observation of adverse effects following HeV vaccination and the perception that HeV vaccination is associated with adverse effects, often of a severe nature, requires further investigation. Public correspondence by Zoetis, the manufacturer of the HeV vaccine, has indicated that reports they have received concerning post-HeV vaccination adverse effects have been uncommon and severe adverse events rare [21]. Validation by veterinarians that illness in horses has been due to HeV vaccination is again rare [22]. These facts would appear to be in contradiction to the reported incidence of adverse effects by respondents in this survey.

There are a number of very strident opponents to HeV vaccination presenting their views via social and other media; their views would appear to have considerable influence on a significant number of horse owners and there appears to be little doubt that their dire warnings and descriptions of adverse post-HeV vaccination events has created a perception amongst horse owners that the vaccine commonly causes adverse effects, often of a serious and sometimes fatal nature [21]. However, the use of social media is similar between HeV vaccinators and non-HeV vaccinators, but HeV vaccinators seem to trust more the advice provided by veterinarians. As veterinarians who have vaccinated many horses against HeV frequently comment that adverse effects are uncommon [22–23], horse owners in close contact with veterinarians are likely to use HeV vaccination.

One cohort of horse owners was identified as more likely to vaccinate their horse or horses against HeV. Horse owners involved in dressage, show jumping, and eventing were more supportive of HeV vaccination than other horse owner classifications. Many owners of pony club horses compete regularly and aggressively but as a generalisation it can be stated that the level of competition for dressage, show jumping and eventing is more intense and horses involved in these sports are likely to be of greater economic value than the those associated with the casual horse owning population, so embracing HeV vaccination represents protection of a significant asset. These horses are more likely to be trained to a higher level of athleticism and therefore owners are more inclined to protect that time commitment. Additionally these horses are more likely to travel further for competition, sometimes being required to stay for multiple day competitions. In these situations the HeV risk of the competition location and other competing horses would be unknown to the owner, giving more reason for vaccination. This cohort of horse owners tend to come from a higher socioeconomic demographic so it could be argued that it is better educated about equine veterinary medicine and therefore more cognizant of the risks associated with HeV and the advantages of vaccination, than is the general horse owning population.

Handling of more than three horses per week compared to only one horse was associated with increased probability of non-vaccination against HeV. It can be suggested that multiple horses are more likely to be kept by riding schools or families for local pleasure riding. These owners might have limited funds to spend on HeV vaccination or may consider their horses to be of less economic importance to invest in vaccination.

As respondents were recruited through horse associations and clubs, we assume that most of these horse associations and club members are horse owners, rather than just horse handlers, with the authority to vaccinate their horses. The survey did not identify if horses were kept in livery stables or yards, or on agistment, or not owned by the responder, either being leased or on loan. Therefore the authors cannot be sure that in all cases, responders had the authority to vaccinate horses to which they had access. Livery stables and yards are not common in Queensland, Australia. Leased or loaned horses are also not commonly encountered in the area surveyed, particularly for those equestrian associations and clubs involved in the survey. Many of the survey responders would own horses’ agisted in paddocks not owned by the responder. Because of the potential legal liability accompanying HeV infection in a livery stable or yard, the majority of these institutions require horses residing on their property to be vaccinated against HeV.

Respondents, who did not vaccinate their horse or horses against HeV, were less likely to vaccinate household pets and were also less likely to be vaccinated against human diseases. Types of pets vaccinated or not vaccinated were not identified in this survey. No questions were asked about respondents philosophical attitudes to vaccination in general.

One concern highlighted by this study was the number of non-vaccinators who consider making money to be a main motivator for veterinarians recommending and conducting HeV vaccinations. This suggests a level of distrust of veterinarians and the veterinary industry as a whole. Alternatively, it may represent a failure of communication between veterinarians and horse owners. Numerous respondents voiced distrust towards Zoetis showing concern that they are financially motivated rather than driven to produce a suitable vaccine that meets horse owner requirements. Due to these concerns it is likely that horse owners would be highly critical of studies produced by Zoetis and it is recommended that independent research be undertaken. Veterinarians who promote HeV vaccination may also be viewed by horse owners in a similar light. When this study was conducted, horses were required to have boosters every six months, but with the annual booster introduced in late 2016, horse owners can halve the number of boosters required. This will help to reduce the cost of vaccination to owners and might revise horse owners’ opinions about the financial motivation of veterinarians.

The role of veterinarians in providing advice to horse owners and not just offering paid services is an important part of the veterinary profession. It had been shown that a higher uptake of HeV risk mitigation practices such as reducing potential contact between bats and horses by keeping horses away from fruiting and flowering trees was associated with more frequent veterinary contact [24].

One of the most significant risk factors identified was the perception by owners that their horses were at low risk of becoming infected with HeV or horse owners were unsure about the HeV risk to their horses. While owners consider that the risk of horses contacting HeV is low, it is likely that the HeV vaccine uptake will remain low. If there was a sudden increase in the incidence of HeV infections, such as occurred in 2011 [9], horse owners are likely to reassess the perceived HeV risk to their horses and might be more inclined to vaccinate. The relatively small number of recent cases may have reduced the level of concern about the risk of horses becoming infected with HeV. However, it is not clear if the limited number of recent cases may be attributable to HeV vaccination or may be due to the sporadic or cyclic nature of the disease. With the current low risk assessment by horse owners it is unlikely that the threat of HeV can be eliminated by HeV horse vaccination alone.

Similarly, severity of HeV infection in humans was underestimated with a significant proportion of horse owners rating it as only moderate compared to severe. There is no definitive treatment for human HeV infection. Inadequate education might be the reason for the misconception held by some horse owners that HeV in humans is a disease of low severity. Perhaps respondents have confused the risk of becoming infected with the level of severity but this seems unlikely and certainly did not appear as an issue in the pilot testing of the questionnaire. Alternatively, horse owners may have been applying an ex post justification to their decisions to not vaccinate by rating the HeV infection in humans as low severity. Recently there has been a debate if a Hendra virus human vaccine should be developed to complement insufficient horse risk management strategies [25–27], such as the limited HeV vaccination of horses and low uptake risk mitigation strategies to reduce spread of HeV from bats to horses [28].

Respondents, who had not vaccinated their horses against HeV, expressed concern about the efficacy of the vaccine, reflecting the obvious argument of ‘why vaccinate if it does not work’. Long-term studies are required to establish the safety, efficacy and appropriate booster vaccination frequency. Such studies would improve public confidence and may lead to improved vaccine uptake.

As expected, membership in horse clubs is predominantly female. The only study reporting on horse ownership in Queensland also noted a greater distribution of female memberships (85% female) [11]. However, gender did not influence the risk of non-vaccination against HeV.

Limitations of this study were that we had no control over whom the equestrian club secretary sends its email to, but we had requested that it be sent to current Queensland members only. Although we had asked the equestrian clubs to specify the number of members to whom emails were sent out with the link to the online survey, no response on this was received from any of the clubs. Hence we were unable to identify the response rate. The aim of this study had been to target horse clubs, including the Thoroughbred and Standardbred racing industries, but unfortunately the racing sector declined involvement. A separate survey of the racing sector would be desirable.

In summary, we identified specific misperceptions about HeV vaccination. Horse owners represent a diverse cross section of the population and therefore it is not surprising they express a wide range of views and knowledge. The survey highlights serious misconceptions concerning the severity of HeV infections in both horses and humans. That fact that a considerable number of responders indicated that HeV infection was not a severe disease in either species is very concerning as it is indicative of a failure by statutory authorities and the veterinary profession to educate horse owners about the true nature of the disease, which possibly has led to a percentage of horse owners rejecting the need for HeV vaccination because they do not regard the disease as a serious risk. The lack of awareness of the potential serious consequences of strangles infection adds support to the conclusion that there is an issue with communication and education, and perhaps trust, between horse owners and veterinarians.

The fact that a significant number of surveyed horse owners’ expressed distrust of the motives of veterinarians is also very concerning. Development of a trust and close relationship of horse owners with veterinarians is the key strategy for the prevention of Hendra virus infection in horses. This important role of veterinarians in facilitating decision-making in all aspects of vaccination, including vaccine implementation has been highlighted in other countries farmers’ perception of the role of veterinary surgeons [29–30]. Distrust creates uncertainty, which can be exploited by others, such as anti-vaccinators, to espouse their own agenda. The release of the HeV vaccine under a minor use permit also created distrust as it was seen by horse owners to be ‘an experimental vaccine’, which was reinforced by word of mouth and social media commentary about frequency and severity of supposed side effects post-vaccination, often fuelled by various parties with specific agenda. Although the vaccine release was expedited for altruistic reasons i.e. protection of humans and horses from a lethal disease, it was seen by many horse owners as a money-making exercise.

The survey demonstrates that underlying public misconceptions and distrust of motives were not adequately addressed prior to release of the HeV vaccine, which despite best intentions accompanying the vaccine’s release, meant that the vaccine uptake was less than expected, and accompanied by considerable horse owner opposition.

## Supporting information

S1 Table Univariate analysis of risk factors associated with no Hendra virus vaccination of horses in 2015 in Queensland, Australia. (DOCX)
