# Supplementary material for: Playing with fire – What is influencing horse owners’ decisions to not vaccinate their horses against deadly Hendra virus infection?
Source: PLoS One. 2017 Jun 21;12(6):e0180062. doi: 10.1371/journal.pone.0180062 (PMC5479593; doi:10.1371/journal.pone.0180062)
Supplement: S1 Table — (DOCX) [file pone.0180062.s001.docx]

**S1 Table: Univariate analysis of risk factors associated with no Hendra virus vaccination of horses in 2015 in Queensland, Australia.**

| **Risk factor** | | | | **Level** | | | **HeV Vac** | | | | | **No HeV Vac** | | | | **OR** | **LCI95%** | **UCI95%** | | **p-value** | **Wald p-value** | | |
| --- | --- | --- | --- | --- | --- | --- | --- | --- | --- | --- | --- | --- | --- | --- | --- | --- | --- | --- | --- | --- | --- | --- | --- |
|  | | | |  | | N | | % | | N % | | | | |  |  |  |  | |  |  | | |
| **HeV infection and vaccination in horses** | | | |  | |  | |  | | |  | |  |  | | |  |  | |  | |  |  |
| Perceived risk of HeV infection in horses (NR=1) | | | | Very high - high | | 73 | | 88.0 | 10 | | | | 12.1 | Ref | | |  |  |  | | <0.001 | |  |
|  | | | | Low - very low | | 134 | | 48.0 | 145 | | | | 52.0 | 7.9 | | | 3.9 | 15.9 | | <0.001 |  | |  |
|  | | | | I don't know | | 7 | | 50.0 | 7 | | | | 50.0 | 7.3 | | | 2.1 | 25.2 | | 0.002 |  | |  |
| Perceived severity of HeV infection in horses (NR=4) | | | | Very severe - severe | | 208 | | 59.1 | 144 | | | | 40.9 | Ref | | |  |  | |  | 0.027 | |  |
|  | | | | Moderate | | 3 | | 33.3 | 6 | | | | 66.7 | 2.9 | | | 0.7 | 11.7 | | 0.138 |  | |  |
|  | | | | Mild to very mild | | 2 | | 20.0 | 8 | | | | 80.0 | 5.8 | | | 1.2 | 27.6 | | 0.028 |  | |  |
|  | | | | I don't know | | 1 | | 20.0 | 4 | | | | 80.0 | 5.8 | | | 0.6 | 52.2 | | 0.118 |  | |  |
| Locality of own horses to previous HeV cases (NR=2) | | | | Within 10-50km | | 76 | | 67.3 | 37 | | | | 32.7 | Ref | | |  |  | |  | 0.007 | |  |
|  | | | | Within 100km | | 54 | | 59.3 | 37 | | | | 40.7 | 1.4 | | | 0.8 | 2.5 | | 0.243 |  | |  |
|  | | | | Within 500km | | 44 | | 54.3 | 37 | | | | 45.7 | 1.7 | | | 1.0 | 3.1 | | 0.068 |  | |  |
|  | | | | More than 500km | | 16 | | 34.8 | 30 | | | | 65.2 | 3.9 | | | 1.9 | 7.9 | | <0.001 |  | |  |
|  | | | | I don't know | | 24 | | 53.3 | 21 | | | | 46.7 | 1.8 | | | 0.9 | 3.6 | | 0.103 |  | |  |
| Ever had adverse reaction to HeV vaccination in own horses? (NR=3) | | | | No | | 153 | | 89.0 | 19 | | | | 11.1 | Ref | | |  |  | |  | <0.001 | |  |
|  | | | | Yes | | 59 | | 58.4 | 42 | | | | 41.6 | 5.7 | | | 3.1 | 10.7 | | <0.001 |  | |  |
|  | | | | Never HeV vaccinated | | 2 | | 1.9 | 101 | | | | 98.1 | 406.7 | | | 92.7 | 1783.8 | | <0.001 |  | |  |
| **HeV infection in humans** | | | |  | |  | |  |  | | | |  |  | | |  |  | |  |  | |  |
| Perceived risk of HeV to people in contact with your horses (NR=2) | | | | Very high - high | | 64 | | 66.0 | 33 | | | | 34.0 | Ref | | |  |  | |  |  | |  |
|  | | | | Low - very low | | 150 | | 53.8 | 129 | | | | 46.2 | 1.7 | | | 1.0 | 2.7 | | 0.037 |  | |  |
| Perceived severity of HeV infection in people (NR=1) | | | | Very severe - severe | | 208 | | 58.8 | 146 | | | | 41.2 | Ref | | |  |  | |  | 0.024 | |  |
|  | | | | Moderate | | 3 | | 23.1 | 10 | | | | 76.9 | 4.8 | | | 1.3 | 17.6 | | 0.020 |  | |  |
|  | | | | Mild - very mild | | 3 | | 33.3 | 6 | | | | 66.7 | 2.9 | | | 0.7 | 11.6 | | 0.143 |  | |  |
| **Tetanus infection and vaccination in horses** | | | |  | |  | |  |  | | | |  |  | | |  |  | |  |  | |  |
| Ever had a horse infected with tetanus? (NR=2) | | | | Yes | | 12 | | 50.0 | 12 | | | | 50.0 | Ref | | |  |  | |  |  | |  |
|  | | | | No | | 202 | | 57.6 | 149 | | | | 42.5 | 0.7 | | | 0.3 | 1.7 | | 0.471 |  | |  |
| Perceived severity of tetanus infection in unvaccinated horses (NR=4) | | | | Very severe - Severe | | 198 | | 58.6 | 140 | | | | 41.4 | Ref | | |  |  | |  | 0.209 | |  |
|  | | | | Moderate | | 11 | | 37.9 | 18 | | | | 62.1 | 2.3 | | | 1.1 | 5.1 | | 0.035 |  | |  |
|  | | | | Mild - very mild | | 3 | | 60.0 | 2 | | | | 40.0 | 0.9 | | | 0.2 | 5.7 | | 0.949 |  | |  |
|  | | | | Not specified | | 2 | | 50.0 | 2 | | | | 50.0 | 1.4 | | | 0.2 | 10.2 | | 0.730 |  | |  |
| Currently vaccinate for tetanus? (NR=2) | | | | Yes | | 182 | | 59.9 | 122 | | | | 40.1 | Ref | | |  |  | |  |  | |  |
|  | | | | No | | 32 | | 44.4 | 40 | | | | 55.6 | 1.9 | | | 1.1 | 3.1 | | 0.018 |  | |  |
| Ever had adverse reaction to a tetanus vaccination in own horses (NR=1) | | | | Yes | | 16 | | 61.5 | 10 | | | | 38.5 | Ref | | |  |  | |  | 0.147 | |  |
|  | | | | No | | 188 | | 58.0 | 136 | | | | 42.0 | 1.2 | | | 0.5 | 2.6 | | 0.727 |  | |  |
|  | | | | I don't vaccinate | | 10 | | 38.5 | 16 | | | | 61.5 | 2.6 | | | 0.8 | 7.8 | | 0.099 |  | |  |
| **Strangles infection and vaccination in horses** | | | |  | |  | |  |  | | | |  |  | | |  |  | |  |  | |  |
| Ever had a horse infected with strangles? (NR=2) | | | | Yes | | 14 | | 38.9 | 22 | | | | 61.1 | Ref | | |  |  | |  |  | |  |
|  | | | | No | | 200 | | 58.8 | 140 | | | | 41.2 | 0.4 | | | 0.2 | 0.9 | | 0.024 |  | |  |
| Perceived severity of strangles infection in unvaccinated horses? (NR=5) | | | | Very severe -severe | | 163 | | 62.0 | 100 | | | | 38.0 | Ref | | |  |  | |  | 0.004 | |  |
|  | | | | Moderate | | 41 | | 46.1 | 48 | | | | 53.9 | 1.9 | | | 1.2 | 3.1 | | 0.009 |  | |  |
|  | | | | Mild - very mild | | 7 | | 33.3 | 14 | | | | 66.7 | 3.3 | | | 1.3 | 8.4 | | 0.014 |  | |  |
|  | | | | Not specified | | 3 | | 100.0 | 0 | | | | 0.0 | 1.0 | | |  |  | |  |  | |  |
| Currently vaccinate for strangles? (NR=6) | | | | Yes | | 169 | | 64.5 | 93 | | | | 35.5 | Ref | | |  |  | |  |  | |  |
|  | | | | No | | 45 | | 39.5 | 69 | | | | 60.5 | 2.8 | | | 1.8 | 4.4 | | <0.001 |  | |  |
| Ever had adverse reaction to strangles vaccination in own horses? (NR=5) | | | | Yes | | 16 | | 55.2 | 13 | | | | 44.8 | Ref | | |  |  | |  | 0.737 | |  |
|  | | | | No | | 195 | | 57.4 | 145 | | | | 42.7 | 0.9 | | | 0.4 | 2.0 | | 0.820 |  | |  |
|  | | | | I don't vaccinate | | 3 | | 42.9 | 4 | | | | 57.1 | 1.6 | | | 0.3 | 8.7 | | 0.560 |  | |  |
| Perceived risk of strangles to horses (NR=1) | | | | Low | | 155 | | 53.5 | 135 | | | | 46.6 | Ref | | |  |  | |  | 0.042 | |  |
|  | | | | High | | 31 | | 66.0 | 16 | | | | 34.0 | 0.6 | | | 0.3 | 1.1 | | 0.112 |  | |  |
|  | | | | I don't know | | 28 | | 71.8 | 11 | | | | 28.2 | 0.5 | | | 0.2 | 0.9 | | 0.034 |  | |  |
| **Other vaccinations by horse owners** | | | |  | |  | |  |  | | | |  |  | | |  |  | |  |  | |  |
| Vaccination of horse owner for common human diseases (NR=3) | | | | Yes | | 182 | | 59.9 | 122 | | | | 40.1 | Ref | | |  |  | |  |  | |  |
|  | | | | No | | 32 | | 44.4 | 40 | | | | 55.6 | 1.9 | | | 1.1 | 3.1 | | 0.018 |  | |  |
| Vaccination of horse owner’s children for common human diseases (NR=4) | | | | Yes | | 120 | | 53.6 | 104 | | | | 46.4 | Ref | | |  |  | |  | 0.167 | |  |
|  | | | | No | | 3 | | 42.9 | 4 | | | | 57.1 | 1.5 | | | 0.3 | 7.0 | | 0.579 |  | |  |
|  | | | | Not applicable | | 91 | | 62.8 | 54 | | | | 37.2 | 0.7 | | | 0.4 | 1.0 | | 0.082 |  | |  |
| Vaccination of horse owner’s pets (NR=6) | | | | Yes | | 199 | | 60.9 | 128 | | | | 39.1 | Ref | | |  |  | |  |  | |  |
|  | | | | No | | 15 | | 30.6 | 34 | | | | 69.4 | 3.5 | | | 1.8 | 6.7 | | 0.000 |  | |  |
| **Perceived motivations of veterinarians to conduct HeV vaccination** (NR=3) | | | | |  |  | |  |  | | | |  |  | | |  |  | |  |  | |  |
| To make money | Unimportant - low important | | | | | 73 | | 81.1 | 17 | | | | 18.9 | Ref | | |  |  | |  | <0.001 | |  |
|  | Neutral | | | | | 53 | | 67.1 | 26 | | | | 32.9 | 2.1 | | | 1.0 | 4.3 | | 0.039 |  | |  |
|  | Important - very important | | | | | 67 | | 38.1 | 109 | | | | 61.9 | 7.0 | | | 3.8 | 12.8 | | <0.001 |  | |  |
|  | Not Specified | | | | | 21 | | 67.7 | 10 | | | | 32.3 | 2.0 | | | 0.8 | 5.1 | | 0.127 |  | |  |
| To protect horses and other animals | Unimportant - low important | | | | | 10 | | 32.3 | 21 | | | | 67.7 | Ref | | |  |  | |  | <0.001 | |  |
|  | Neutral | | | | | 9 | | 20.0 | 36 | | | | 80.0 | 1.9 | | | 0.7 | 5.4 | | 0.229 |  | |  |
|  | Important - very important | | | | | 186 | | 66.4 | 94 | | | | 33.6 | 0.2 | | | 0.1 | 0.5 | | <0.001 |  | |  |
|  | Not Specified | | | | | 9 | | 45.0 | 11 | | | | 55.0 | 0.6 | | | 0.2 | 1.9 | | 0.360 |  | |  |
| To protect horse owners | Unimportant - low important | | | | | 10 | | 30.3 | 23 | | | | 69.7 | Ref | | |  |  | |  | <0.001 | |  |
|  | Neutral | | | | | 9 | | 17.3 | 43 | | | | 82.7 | 2.1 | | | 0.7 | 5.8 | | 0.165 |  | |  |
|  | Important - very important | | | | | 183 | | 69.9 | 79 | | | | 30.2 | 0.2 | | | 0.1 | 0.4 | | <0.001 |  | |  |
|  | Not Specified | | | | | 12 | | 41.4 | 17 | | | | 58.6 | 0.6 | | | 0.2 | 1.8 | | 0.365 |  | |  |
| To protect the general public | Unimportant - low important | | | | | 14 | | 35.0 | 26 | | | | 65.0 | Ref | | |  |  | |  | 0.000 | |  |
|  | Neutral | | | | | 13 | | 22.8 | 44 | | | | 77.2 | 1.8 | | | 0.7 | 4.5 | | 0.190 |  | |  |
|  | Important - very important | | | | | 175 | | 70.3 | 74 | | | | 29.7 | 0.2 | | | 0.1 | 0.5 | | <0.001 |  | |  |
|  | Not Specified | | | | | 12 | | 40.0 | 18 | | | | 60.0 | 0.8 | | | 0.3 | 2.1 | | 0.669 |  | |  |
| To protect themselves and their family | Unimportant - low important | | | | | 5 | | 35.7 | 9 | | | | 64.3 | Ref | | |  |  | |  | <0.001 | |  |
|  | Neutral | | | | | 2 | | 9.1 | 20 | | | | 90.9 | 5.6 | | | 0.9 | 34.2 | | 0.065 |  | |  |
|  | Important - very important | | | | | 204 | | 60.6 | 122 | | | | 37.4 | 0.3 | | | 0.1 | 1.0 | | 0.053 |  | |  |
|  | | Not Specified | | | | 3 | | 21.4 | 11 | | | | 78.6 | 2.0 | | | 0.4 | 10.9 | | 0.407 |  | |  |
| **Horse riding activities conducted by horse owner** (NR=0) | | | |  | |  | |  |  | | | |  |  | | |  |  | |  |  | |  |
| Carriage | | | | No | | 212 | | 57.3 | 158 | | | | 42.7 | Ref | | |  |  | |  |  | |  |
|  | | | | Yes | | 2 | | 33.3 | 4 | | | | 66.7 | 2.7 | | | 0.5 | 14.8 | | 0.258 |  | |  |
| Breeding | | | | No | | 167 | | 61.6 | 104 | | | | 38.4 | Ref | | |  |  | |  |  | |  |
|  | | | | Yes | | 47 | | 44.8 | 58 | | | | 55.2 | 2.0 | | | 1.3 | 3.1 | | 0.003 |  | |  |
| Dressage/Show jumping/Eventing | | | | No | | 83 | | 45.4 | 100 | | | | 54.6 | Ref | | |  |  | |  |  | |  |
|  | | | | Yes | | 131 | | 67.9 | 62 | | | | 32.1 | 0.4 | | | 0.3 | 0.6 | | <0.001 |  | |  |
| Show/Pony club | | | | No | | 155 | | 61.0 | 99 | | | | 39.0 | Ref | | |  |  | |  |  | |  |
|  | | | | Yes | | 59 | | 48.4 | 63 | | | | 51.6 | 1.7 | | | 1.1 | 2.6 | | 0.021 |  | |  |
| Pleasure/Trail riding | | | | No | | 120 | | 60.9 | 77 | | | | 39.1 | Ref | | |  |  | |  |  | |  |
|  | | | | Yes | | 94 | | 52.5 | 85 | | | | 47.5 | 1.4 | | | 0.9 | 2.1 | | 0.101 |  | |  |
| Western/Camp drafting/Cutting/Stockhorse | | | | No | | 161 | | 60.8 | 104 | | | | 39.3 | Ref | | |  |  | |  |  | |  |
|  | | | | Yes | | 53 | | 47.8 | 58 | | | | 52.3 | 1.7 | | | 1.1 | 2.6 | | 0.021 |  | |  |
| Polo/Polocrosse | | | | No | | 208 | | 58.1 | 150 | | | | 41.9 | Ref | | |  |  | |  |  | |  |
|  | | | | Yes | | 6 | | 33.3 | 12 | | | | 66.7 | 2.8 | | | 1.0 | 7.6 | | 0.046 |  | |  |
| Racing | | | | No | | 213 | | 57.3 | 159 | | | | 42.7 | Ref | | |  |  | |  |  | |  |
|  | | | | Yes | | 1 | | 25.0 | 3 | | | | 75.0 | 4.0 | | | 0.4 | 39.0 | | 0.230 |  | |  |
| Endurance | | | | No | | 208 | | 57.5 | 154 | | | | 42.5 | Ref | | |  |  | |  |  | |  |
|  | | | | Yes | | 6 | | 42.9 | 8 | | | | 57.1 | 1.8 | | | 0.6 | 5.3 | | 0.285 |  | |  |
| Number of activities that own horses performing | | | | 1 | | 88 | | 63.8 | 50 | | | | 36.2 | Ref | | |  |  | |  | 0.013 | |  |
|  | | | | 2 | | 82 | | 59.9 | 55 | | | | 40.2 | 1.2 | | | 0.7 | 1.9 | | 0.500 |  | |  |
|  | | | | 3 | | 31 | | 46.3 | 36 | | | | 53.7 | 2.0 | | | 1.1 | 3.7 | | 0.018 |  | |  |
|  | | | | >3 | | 13 | | 38.2 | 21 | | | | 61.8 | 2.9 | | | 1.3 | 6.2 | | 0.008 |  | |  |
| **Horse management** | | | |  | |  | |  |  | | | |  |  | | |  |  | |  |  | |  |
| Number of horses owned and frequently handled on property (NR=3) | | | | 1 | | 35 | | 79.6 | 9 | | | | 20.5 | Ref | | |  |  | |  | <0.001 | |  |
|  | | | | 2-3 | | 87 | | 64.9 | 47 | | | | 35.1 | 2.1 | | | 0.9 | 4.7 | | 0.074 |  | |  |
|  | | | | >3 | | 92 | | 46.5 | 106 | | | | 53.5 | 4.5 | | | 2.0 | 9.8 | | <0.001 |  | |  |
| Number of other horses on property (not owned) (NR=6) | | | | 0 | | 136 | | 53.8 | 117 | | | | 46.3 | Ref | | |  |  | |  | 0.116 | |  |
|  | | | | 1-10 | | 55 | | 60.4 | 36 | | | | 39.6 | 0.8 | | | 0.5 | 1.2 | | 0.272 |  | |  |
|  | | | | >10 | | 23 | | 71.9 | 9 | | | | 28.1 | 0.5 | | | 0.2 | 1.0 | | 0.056 |  | |  |
| Frequency of own horses going to locations where they interact with other horses (NR=3) | | | | Daily/weekly | | 86 | | 59.7 | 58 | | | | 40.3 | Ref | | |  |  | |  | 0.126 | |  |
|  | | | | Monthly | | 94 | | 57.0 | 71 | | | | 43.0 | 1.1 | | | 0.7 | 1.8 | | 0.625 |  | |  |
|  | | | | Yearly | | 27 | | 61.4 | 17 | | | | 38.6 | 0.9 | | | 0.5 | 1.9 | | 0.846 |  | |  |
|  | | | | Never | | 5 | | 26.3 | 14 | | | | 73.7 | 4.2 | | | 1.4 | 12.2 | | 0.009 |  | |  |
|  | | | | Unknown | | 2 | | 50.0 | 2 | | | | 50.0 | 1.5 | | | 0.2 | 10.8 | | 0.698 |  | |  |
| Frequency of new horses entering own property (NR=2) | | | | Daily/weekly | | 15 | | 62.5 | 9 | | | | 37.5 | Ref | | |  |  | |  | 0.887 | |  |
|  | | | | Monthly | | 31 | | 57.4 | 23 | | | | 42.6 | 1.2 | | | 0.5 | 3.3 | | 0.673 |  | |  |
|  | | | | Yearly | | 82 | | 54.3 | 69 | | | | 45.7 | 1.4 | | | 0.6 | 3.4 | | 0.454 |  | |  |
|  | | | | Never | | 69 | | 57.5 | 51 | | | | 42.5 | 1.2 | | | 0.5 | 3.0 | | 0.651 |  | |  |
|  | | | | Unknown | | 17 | | 62.7 | 10 | | | | 37.0 | 1.0 | | | 0.3 | 3.1 | | 0.973 |  | |  |
| On a weekly basis, how many people interact with your horse/s? (NR=0) | | | | Nil | | 4 | | 66.7 | 2 | | | | 33.3 | Ref | | |  |  | |  | 0.982 | |  |
|  | | | | 1-5 | | 185 | | 56.8 | 141 | | | | 43.3 | 1.5 | | | 0.3 | 8.4 | | 0.629 |  | |  |
|  | | | | 6-10 | | 16 | | 59.3 | 11 | | | | 40.7 | 1.4 | | | 0.2 | 8.9 | | 0.738 |  | |  |
|  | | | | >10 | | 8 | | 53.3 | 7 | | | | 46.7 | 1.8 | | | 0.2 | 12.6 | | 0.579 |  | |  |
|  | | | | Don't Know | | 1 | | 50.0 | 1 | | | | 50.0 | 2.0 | | | 0.1 | 51.6 | | 0.676 |  | |  |
| Number of years of horse ownership (NR=2) | | | | <5 | | 19 | | 73.1 | 7 | | | | 26.9 | Ref | | |  |  | |  | 0.114 | |  |
|  | | | | 6-10 | | 24 | | 70.6 | 10 | | | | 29.4 | 1.1 | | | 0.4 | 3.5 | | 0.832 |  | |  |
|  | | | | 11-15 | | 16 | | 55.2 | 13 | | | | 44.8 | 2.0 | | | 0.7 | 6.9 | | 0.172 |  | |  |
|  | | | | >15 | | 155 | | 54.4 | 130 | | | | 45.6 | 2.3 | | | 0.9 | 5.6 | | 0.072 |  | |  |
|  | | | | Unknown | | 0 | | 0.0 | 2 | | | | 100.0 | 1.0 | | |  |  | |  |  | |  |
| Frequency of having own horses' teeth checked (NR=1) | | | | Yearly | | 141 | | 64.1 | 79 | | | | 35.9 | Ref | | |  |  | |  | 0.005 | |  |
|  | | | | 1-2 years | | 64 | | 48.5 | 68 | | | | 51.5 | 1.9 | | | 1.2 | 2.9 | | 0.004 |  | |  |
|  | | | | >2 years or never | | 9 | | 40.9 | 13 | | | | 59.1 | 2.6 | | | 1.1 | 6.3 | | 0.038 |  | |  |
|  | | | | Don't Know | | 0 | | 0.0 | 2 | | | | 100.0 | 1.0 | | |  |  | |  |  | |  |
| Frequency of deworming own horses (NR=0) | | | | < 3months | | 177 | | 59.4 | 121 | | | | 40.6 | Ref | | |  |  | |  | 0.132 | |  |
|  | | | | 4-6 months | | 28 | | 50.0 | 28 | | | | 50.0 | 1.5 | | | 0.8 | 2.6 | | 0.193 |  | |  |
|  | | | | > 6 months | | 9 | | 40.9 | 13 | | | | 59.1 | 2.1 | | | 0.9 | 5.1 | | 0.096 |  | |  |
| Size of property where own horses are kept (NR=3) | | | | < 5 acres | | 44 | | 63.8 | 25 | | | | 36.2 | Ref | | |  |  | |  | 0.032 | |  |
|  | | | | 6-10 acres | | 62 | | 62.6 | 37 | | | | 37.4 | 1.1 | | | 0.6 | 2.0 | | 0.880 |  | |  |
|  | | | | 11-50 acres | | 62 | | 57.9 | 45 | | | | 42.1 | 1.3 | | | 0.7 | 2.4 | | 0.441 |  | |  |
|  | | | | >50 acres | | 44 | | 44.4 | 55 | | | | 55.6 | 2.2 | | | 1.2 | 4.1 | | 0.014 |  | |  |
|  | | | | Unknown | | 2 | | 100.0 | 0 | | | | 0.0 | 1.0 | | |  |  | |  |  | |  |
| **Ownership of other animals by horse owner** (NR=2) | | | |  | |  | |  |  | | | |  |  | | |  |  | |  |  | |  |
| Cats | | | | Yes | | 98 | | 59.0 | 68 | | | | 41.0 | Ref | | |  |  | |  |  | |  |
|  | | | | No | | 116 | | 55.2 | 94 | | | | 44.8 | 1.2 | | | 0.8 | 1.8 | | 0.460 |  | |  |
| Dogs | | | | Yes | | 15 | | 60.0 | 10 | | | | 40.0 | Ref | | |  |  | |  |  | |  |
|  | | | | No | | 199 | | 56.7 | 152 | | | | 43.3 | 1.1 | | | 0.5 | 2.6 | | 0.747 |  | |  |
| Cattle | | | | Yes | | 21 | | 52.5 | 19 | | | | 47.5 | Ref | | |  |  | |  |  | |  |
|  | | | | No | | 193 | | 57.4 | 143 | | | | 42.6 | 0.8 | | | 0.4 | 1.6 | | 0.551 |  | |  |
| Sheep | | | | Yes | | 53 | | 49.1 | 55 | | | | 50.9 | Ref | | |  |  | |  |  | |  |
|  | | | | No | | 161 | | 60.1 | 107 | | | | 39.9 | 0.6 | | | 0.4 | 1.0 | | 0.052 |  | |  |
| Goats | | | | Yes | | 60 | | 53.6 | 52 | | | | 46.4 | Ref | | |  |  | |  |  | |  |
|  | | | | No | | 154 | | 58.3 | 110 | | | | 41.7 | 0.8 | | | 0.5 | 1.3 | | 0.394 |  | |  |
| Alpacas | | | | Yes | | 61 | | 50.0 | 61 | | | | 50.0 | Ref | | |  |  | |  |  | |  |
|  | | | | No | | 153 | | 60.2 | 101 | | | | 39.8 | 0.7 | | | 0.4 | 1.0 | | 0.061 |  | |  |
| Other grazing animals | | | | Yes | | 71 | | 52.2 | 65 | | | | 47.8 | Ref | | |  |  | |  |  | |  |
|  | | | | No | | 143 | | 59.6 | 97 | | | | 40.4 | 0.7 | | | 0.5 | 1.1 | | 0.166 |  | |  |
| **Demographic information of horse owner** | | | |  | |  | |  |  | | | |  |  | | |  |  | |  |  | |  |
| Age (NR=3) | | | | Under 30 | | 47 | | 61.0 | 30 | | | | 39.0 | Ref | | |  |  | |  | 0.167 | |  |
|  | | | | 31-40 | | 42 | | 50.6 | 41 | | | | 49.4 | 1.5 | | | 0.8 | 2.9 | | 0.185 |  | |  |
|  | | | | 41-50 | | 57 | | 52.3 | 52 | | | | 47.7 | 1.4 | | | 0.8 | 2.6 | | 0.237 |  | |  |
|  | | | | Over> | | 68 | | 64.2 | 38 | | | | 35.4 | 0.9 | | | 0.5 | 1.6 | | 0.667 |  | |  |
|  | | | | Unknown | | 0 | | 0.0 | 1 | | | | 100.0 | 1.0 | | |  |  | |  |  | |  |
| Gender (NR=1) | | | | Male | | 9 | | 60.0 | 6 | | | | 40.0 | Ref | | |  |  | |  | 0.709 | |  |
|  | | | | Female | | 204 | | 57.0 | 154 | | | | 43.0 | 1.1 | | | 0.4 | 3.2 | | 0.817 |  | |  |
|  | | | | Other | | 1 | | 33.3 | 2 | | | | 66.7 | 3.0 | | | 0.2 | 40.9 | | 0.410 |  | |  |
| Highest level of education (NR=6) | | | | Year 10 | | 20 | | 57.1 | 15 | | | | 42.9 | Ref | | |  |  | |  | 0.420 | |  |
|  | | | | Year 12 | | 41 | | 50.6 | 40 | | | | 49.4 | 1.3 | | | 0.6 | 2.9 | | 0.519 |  | |  |
|  | | | | Trade/diploma | | 47 | | 52.8 | 42 | | | | 47.2 | 1.2 | | | 0.5 | 2.6 | | 0.663 |  | |  |
|  | | | Undergraduate degree | | | 58 | | 65.9 | 30 | | | | 34.1 | 0.7 | | | 0.3 | 1.5 | | 0.364 |  | |  |
|  | | | Postgraduate degree | | | 45 | | 58.4 | 32 | | | | 41.6 | 0.9 | | | 0.4 | 2.1 | | 0.897 |  | |  |
|  | | | Other | | | 3 | | 50.0 | 3 | | | | 50.0 | 1.3 | | | 0.2 | 7.6 | | 0.745 |  | |  |

NR = No Responses
